# Probiotic effects on skin health: comprehensive visual analysis and perspectives

**DOI:** 10.3389/fmicb.2024.1453755

**Published:** 2024-12-03

**Authors:** Kexin Deng, Xiaofei Fan, Zhigen Yuan, Dian Li

**Affiliations:** ^1^Department of Orthopaedics, The Second People's Hospital of Hunan Province (Brain Hospital of Hunan Province), Changsha, Hunan, China; ^2^Department of Plastic and Reconstruction, The Third Xiangya Hospital of Central South University, Changsha, Hunan, China; ^3^Shandong Medical College, Jinan, Shandong, China; ^4^Department of Epidemiology, Los Angeles Fielding School of Public Health, University of California, Los Angeles, Los Angeles, CA, United States

**Keywords:** prebiotics, bibliometrics, skin, probiotics, bacteria

## Abstract

**Background:**

Bacteria play a crucial role in maintaining the health of human skin. Research has demonstrated that probiotics present notable benefits for extraintestinal organs. Despite the extensive research on the impact of probiotics on skin health, there is a notable absence of regulatory frameworks governing their external application, with no approval from the FDA for any probiotic products for external use. The aim of this study is to offer a thorough summary of the research status in the field since 2000 and project future trends.

**Method:**

The Web of Science Core Collection and SCI-Expanded index were selected for an extensive search of studies concerning the role of probiotics in skin health since 2000. A total of 1,306 publications were identified. Employing a double-blind method, two subsets of literature were scrutinized and subsequently combined for analysis. Using CiteSpace, this research explored key aspects such as primary countries, institutions, authors, journals, trending topics, research frontiers, and emerging patterns in research related to application of probiotic for skin health.

**Result:**

This article included 709 research papers. The number of published papers has shown a rapid increase. The United States had the highest number of research papers (128), and Canada had the highest intermediate centrality (0.23). The University of California System emerged as the most prolific institution. Huang, Chun-Ming has published the most articles, and his research is at the forefront among those prolific authors. Twelve clusters were identified, with cluster #0 skin microbiota, #3 mechanisms, and #8 antimicrobial being the most recent. As for the hot topic, “diversity,” “health,” “skin microbiome,” “oxidative stress,” “microbiota,” and “antioxidants” have been at the forefront of the current field. The overall research trend has shifted from clinical trials to mechanistic exploration and from oral treatments to external applications, with the research level moving from general categories to specific strains.

**Conclusion:**

This paper summarized and visualized academic achievements in the field of probiotic application for skin health using CiteSpace and VOSviewer, offering a systematic and comprehensive perspective, along with a longitudinal overview of this research field.

## 1 Introduction

Immediately after birth, the human body establishes an indissoluble connection with bacteria residing on its surface (Grice and Segre, 2011). These microorganisms coexist symbiotically with the host, crucially contributing to the preservation of the host's normal physiological and immune functions (Grice and Segre, 2011). In the absence of bacterial presence on the skin, as observed in sterile animals, the skin becomes more vulnerable to chemical toxicity, pathogenic infections, impaired tissue regeneration, and various other health-related issues (Shavandi et al., 2020). These factors highlight the essential role of skin microflora in maintaining skin health (Shavandi et al., 2020). In 2013, experts outlined a precise definition and delineation of the term “probiotics” as living microorganisms that, when administered in suitable amounts, elicit beneficial effects on the host's health (Hill et al., 2014). Probiotics possess the capacity to exert a positive impact on health and play a role in the prevention of aging (AT and Action, 1999). Prebiotics have the ability to selectively promote the growth and functionality of probiotics, leading to advantages for the host and contributing to improved health (Roberfroid et al., 2010). Recently, there has been an increasing focus on non-living preparations of microorganisms and/or their components, collectively termed “post-biotics.” According to the International Scientific Association of Probiotics and Prebiotics (ISAPP), post-biotics are defined as “preparations of inanimate microorganisms and/or their components that confer a health benefit on the host.” These include inactivated bacterial cells, their cell fragments, and components such as cell wall structures or membrane lipids. These have demonstrated efficacy in supporting various health functions, including decreasing pathogen resistance to antibiotics, lowering inflammatory markers, and enhancing skin health (Vinderola et al., 2024). Both probiotics and post-biotics may play crucial roles in maintaining skin health. Studies suggest that imbalances in the skin's microbiota can lead to a range of skin diseases, such as acne, rosacea, and eczema (Zólkiewicz et al., 2020). Numerous studies have demonstrated the beneficial role of probiotics in maintaining skin health. Probiotics can assist in maintaining and enhancing the equilibrium of the skin's microbiota, thereby contributing to the prevention and amelioration of diverse skin conditions (Sinha et al., 2021). Incorporating probiotic products into one's diet or applying them topically can enhance the diversity and equilibrium of the skin microbiota (Truglio et al., 2024; Whiting et al., 2024; Buhas, et al., 2023). Meanwhile, probiotics have been demonstrated to mitigate skin inflammation and oxidative stress, consequently enhancing the skin's barrier function and lowering the susceptibility to skin infections (Miao et al., 2024; Yin et al., 2024).

The United States Food and Drug Administration (FDA) categorizes probiotics into distinct categories based on the type of products, which include dietary supplements, food items, food additives, cosmetics, or pharmaceutical drugs. However, it is worth noting that currently, there are no specific regulations or laws governing live probiotics intended for external use, and the FDA has not yet approved any live probiotic products designed for external application. This highlights the pressing need to establish regulations and guidelines for such products (Lee et al., 2019). The main objective of this paper is to conduct a thorough evaluation of the present status within the research domain of probiotic application for skin health. Through a meticulous review of existing scientific literature, this study aimed to provide valuable insights and data that can serve as a reference for formulating relevant laws and regulations in alignment with the advancements in scientific knowledge in this field.

Bibliometric analysis is a quantitative method used to investigate, examine, and analyze research outcomes within a specific field (Mayr and Scharnhorst, 2015). The primary objective of literature analysis is to acquire detailed information such as authors, keywords, references, institutions, countries, etc. (Abramo et al., 2011). This information can then be further analyzed for property analysis and performance evaluation to assess the progress and developments within specific fields (Abramo et al., 2011). Ma and Xi emphasized that utilizing the co-citation visual measurement method in bibliometrics is optimal for interpreting data. This method is favored for providing more reliable and comprehensive results compared to other approaches (Ma and Xi, 1992).

The paper aimed to offer readers a thorough and systematic scientometric study about the role of probiotics in skin health. CiteSpace, a visualization tool, was used to analyze the citations obtained from the Web of Science Core Collection (WoSCC) (Borner et al., 2003). Utilizing this software can offer effective support for scientometric work in the field, addressing the knowledge gaps in bibliometric reviews about this topic. The primary objectives of this study were (1) to summarize research related to the application of probiotics for skin health since the 21^st^ century in the context of globalization, (2) to examine the trending research topics and their characteristics in this field, and (3) to analyze emerging research directions with potential value based on trend analysis.

## 2 Method and data

### 2.1 Research method

Bibliometrics, as a subfield of informatics, provides a highly objective and reliable method for evaluating achievements within a specific field. By systematically analyzing large amounts of information, it can effectively clarify the research trajectory, categorize research themes, detect changes in research directions, and conduct performance analyses at different levels, such as countries, institutions, authors, and so on (Abramo et al., 2011). This helps in understanding the landscape of research in a particular area and identifying key contributors and trends (Donthu et al., 2021). The use of modern digital and visualization analysis techniques enhances the timeliness, accessibility, and reproducibility of bibliometric research (van Eck and Waltman, 2017).

CiteSpace, developed by Dr. Chaomei Chen at Drexel University, is an information visualization software based on citation analysis, used to explore the structure and distribution of scientific knowledge (Chen, 2006). It utilizes methods such as co-citation, co-occurrence, burst detection, and cluster analysis to examine theoretical perspectives, development trends, and research hotspots (Chen, 2006; Hou et al., 2018; Su and Lee, 2010; Pan et al., 2018). Co-citation analysis investigates the co-citation relationship between studies, where frequent co-citations imply higher similarity (Hou et al., 2018). Co-occurrence analysis calculates the frequency of keyword co-occurrences to measure their affinity (Su and Lee, 2010). Burst detection identifies changes in the frequency of specific keywords, and cluster analysis groups objects based on similarity to generate and analyze clusters (Pan et al., 2018). Centrality is a crucial indicator of a node's significance, with nodes showing intermediate centrality >0.1 defined as key nodes, which often serve as bridges connecting different research entities, such as articles, keywords, and countries (Chen, 2006). CiteSpace is particularly valuable for identifying critical points and future research trends, which is why it was used in this study (Pan et al., 2018).

### 2.2 Data resource

**Figure 1:**
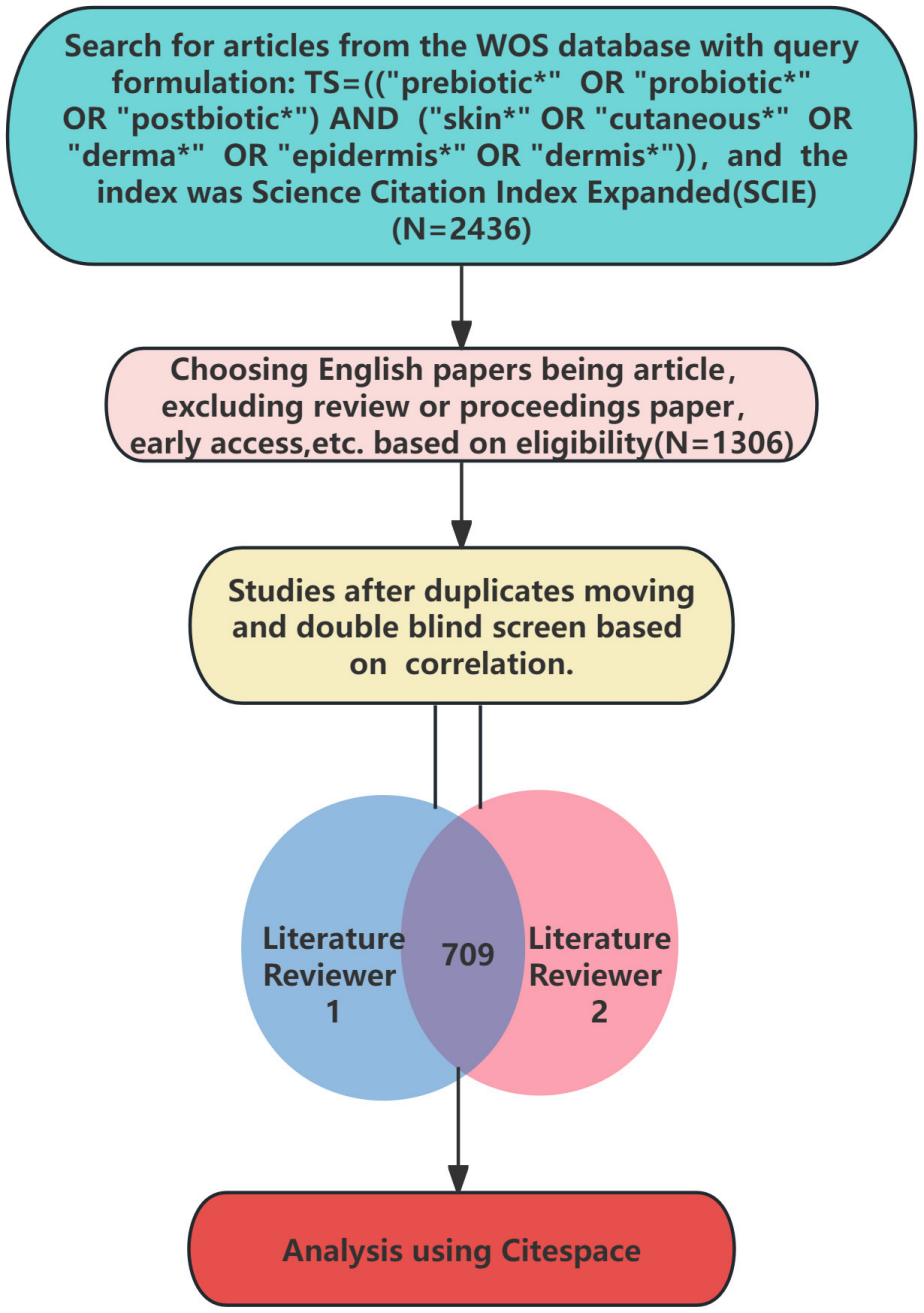
The search strategy used for the present bibliometric analysis.

In this paper, Web of Science Core Collection (WoSCC) was selected and the index was Science Citation Index Expanded (SCIE). The retrieval formula in this paper was as follows: TS = ((“prebiotic^*^” OR “probiotic^*^” OR “postbiotic^*^”) AND (“skin^*^” OR “cutaneous^*^” OR “derma^*^” OR “epidermis^*^” OR “dermis^*^”)). The time span was from January 1^st^, 2000 to December 31^st^, 2023. The initial search yielded a total of 1,603 English articles being the type of “article.” Following a double-blinded screening process and subsequent merging of the two selected subsets, a final selection of 709 non-duplicate articles was made. During the double-blinded screening process, the following criteria were applied: (1) The research subjects must be mammals, with amphibians, fish, insects, mollusks, etc., being excluded; (2) The research must involve the skin or areas indirectly related to the skin; (3) The study should encompass known probiotics or other commensal microorganisms on the human body; (4) The articles must adhere to standard English writing conventions. The literature retrieval and screening process is recorded in Figure 1.

## 3 Results

### 3.1 Analysis of publishing trend

**Figure 2:**
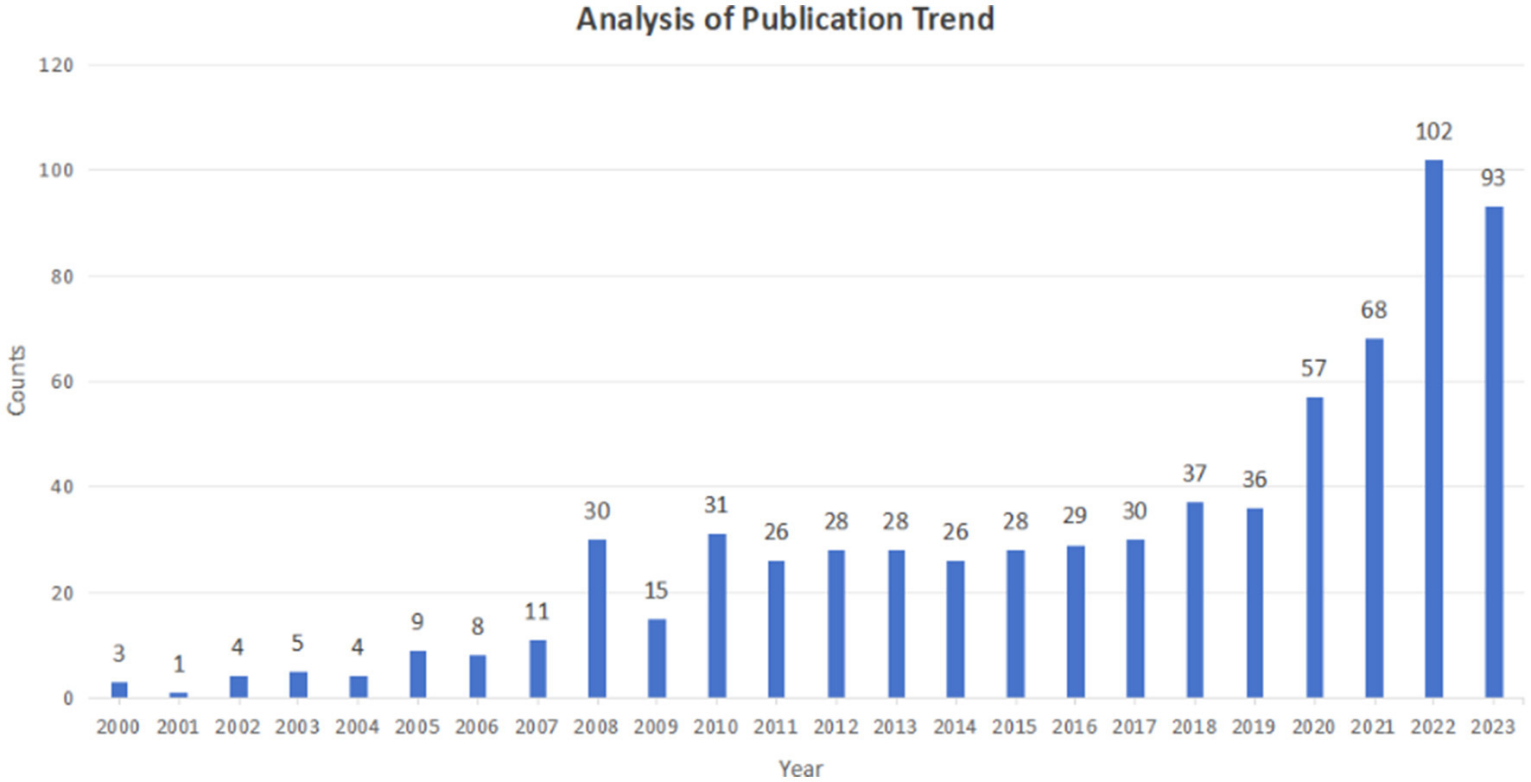
Time evolution of the total number of publications per year in the WOS database. The ordinate represents the number of published papers, while the abscissa represents the years.

The publication trend in academic literature plays a vital role in assessing the progress and development of research within a specific field. Figure 2 depicts the annual distribution of articles related to the application of probiotics about skin health on Web of Science since 2000. In recent years, there has been significant growth and advancement in the study related to the role of probiotics in skin health, as evidenced by the steady increase in the number of articles related to this topic. Particularly noteworthy is the upward trend observed in the last 3 years, with an average annual article count reaching 88. Prior to 2020, the average annual article count was relatively lower, at around 19 articles per year. However, there has been a noticeable surge in publications post-2019. While the volume of research has not yet peaked, the field of this topic is rapidly progressing. Additionally, based on the current research trends, there appears to be significant potential for growth in this field in the upcoming years, with continued expansion and advancement anticipated.

#### 3.1.1 National analysis

**Figure 3:**
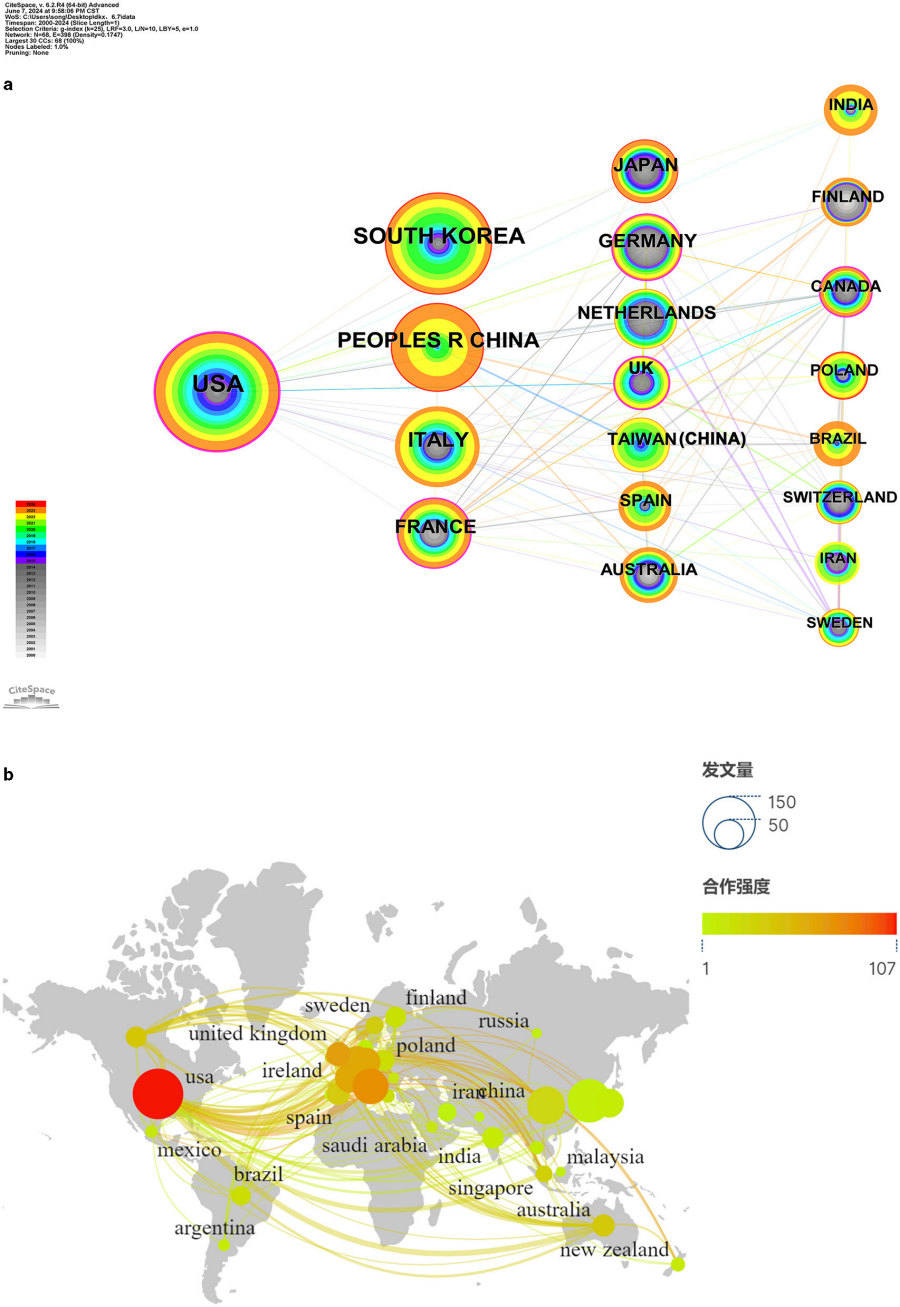
Analysis of publications and collaborations among countries. **(a)** Cooperation network of countries. The “Time Slicing” was set to “2000–2023” with the “Years Per Slice” being 1, and the threshold was set to the top “20.” The size of the nodes corresponds to the volume of publications. **(b)** Analysis of geographical distribution of publications, which was created by connected CiteSpace with Google Maps. To improve the visual appearance, connections between countries with fewer collaborations were left out.

The quantitative analysis conducted at the country level not only helped in identifying the core countries in the field of probiotic applications for skin health but also revealed patterns of academic communication and collaboration among nations. This analysis produced a national network map with 68 nodes, 398 connections, and a density of 0.1747, as depicted in Figure 3a. The thickness of the purple rings in the figure indicated the degree of intermediary centrality, with values >0.1, reflecting the importance of a country's position within the network. A higher intermediary centrality suggests that the node plays a stronger bridging role connecting other nodes.

The top 20 countries in terms of their involvement in research related to probiotic applications for skin health are listed in Table 1. The “Frequency” column shows the number of publications from each country, while the “Centrality” column indicates the intermediary centrality within the field. Figure 3a demonstrates that countries with more international connections tend to exhibit higher centrality and possess greater authority within the research network.

**Table 1:** The centrality and counts of literature in the top 10 countries.

**Rank**	**Country**	**Frequency**	**Centrality**	**Years**
1	USA	128	0.22	2002
2	South Korea	95	0	2006
3	Peoples R China	71	0	2014
4	Italy	64	0.06	2002
5	France	44	0.14	2002
6	Japan	42	0.05	2007
7	Germany	41	0.12	2001
8	Netherlands	34	0.14	2005
9	England	29	0.16	2006
10	Taiwan	29	0.01	2012
11	Spain	27	0.03	2003
12	Australia	25	0.08	2000
13	India	23	0	2007
14	Finland	22	0.01	2000
15	Canada	22	0.23	2003
16	Poland	21	0.08	2009
17	Brazil	19	0.03	2002
18	Switzerland	19	0.09	2006
19	Iran	17	0	2011
20	Sweden	16	0.03	2004

In Table 1, the key countries could be found in the field of probiotic applications for skin health. The United States was considered as the leading country in probiotic research related to skin health due to its highest publication count of 128 and second-ranking position in intermediary centrality at 0.22. This indicated the United States' strong presence in the field, showcasing excellence in research output and international collaborations, thus solidifying its dominant position.

Canada, ranking first in the intermediary centrality, emphasized international cooperation but with only 22 publications, demonstrating its crucial role as a bridge builder in the research network. South Korea followed closely behind the United States with 95 publications, while China ranked third with 71 articles. However, their low intermediary centrality values suggest a lack of emphasis on international cooperation, potentially hindering high-quality and extensive research endeavors. Countries like India and Iran also displayed low intermediary centrality, indicating a similar pattern of the limited international engagement. Australia and Finland were among the earliest countries to delve into research about probiotic applications for skin health.

Moreover, France, Germany, Sweden, and the United Kingdom surpassed an intermediary centrality of 0.1 and exhibited substantial publication counts, signaling strong international collaborations in these nations. Figure 3b illustrated that research efforts were mainly concentrated in Europe, North America, and some East Asian countries. The countries with intermediary centrality exceeding 0.1 were predominantly developed nations in Europe and North America, underlining their leadership roles in the field and highlighting their emphasis on research through global partnerships.

Overall, the data suggests that developed countries have taken a leading position in probiotic research related to skin health, emphasizing collaborative efforts on an international scale to drive advancements in the field.

#### 3.1.2 Institutional analysis

**Figure 4:**
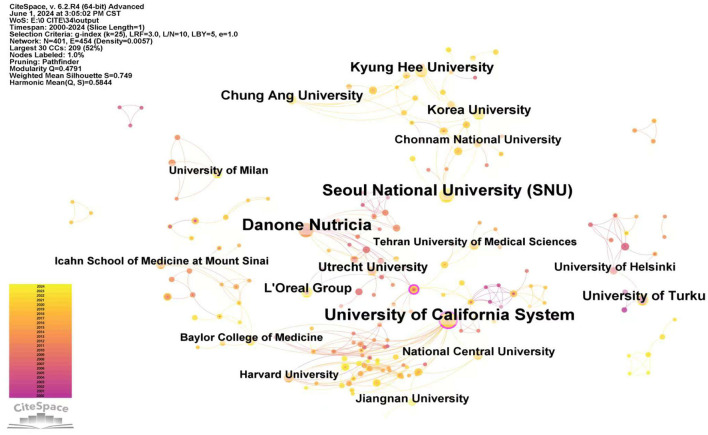
Interinstitutional cooperation network, by using “Institution” in CiteSpace as the analysis object, the map with a density of 0.0057, comprising 407 network nodes and 454 connections was obtained. Institutions with a purple outer ring indicate a centrality >0.1. The size of the nodes corresponds to the number of article publications by each institution. To enhance the visual appearance, the lines between institutions with relatively low collaborations have been omitted. The different colors represent distinct years, with each color corresponding to 1 year from 2024 (top) to 2000 (bottom), as indicated in the legend.

Figure 4 displayed relatively dense nodes. This indicated that institutional cooperation in the field of skin health and probiotic was relatively frequent, with most research institutions engaged in collaborative efforts. Some of these institutional collaborations exhibited regional characteristics, emphasizing the importance of maintaining inter-institutional researches to promote academic exchanges within the field.

Additionally, this study compiled a list of the top 20 institutions involved in the application of probiotics for skin health. Table 2 presented an overview of these institutions. The University of California System led the list with 29 publications, surpassing other institutions. It has also been one of the only two institutions with a intermediary centrality exceeding 0.1, the other being the Institut National de la Santé et de la Recherche Médicale (Inserm). Although the latter had a intermediary centrality of 0.12, its number of publications was relatively low, with only 5 articles. Danone Nutricia and L'Oreal Group are a specialized infant nutrition company and a skincare company, respectively. They not only initiated research in this field earlier but also made significant contributions and engaged in extensive international collaboration. The excellent performances of these commercial companies highlight the tremendous potential for translating academic achievements in the fields of probiotics application for skin health into direct economic impact.

**Table 2:** The top 20 institutions of publication counts.

**Institutions**	**Frequency**	**First publication years**	**Intermediary centrality**
University of California System	29	2013	0.13
Danone Nutricia	19	2004	0.06
Seoul National University (SNU)	18	2009	0
Chung Ang University	18	2012	0
Kyung Hee University	11	2015	0
University of Turku	10	2000	0
Korea University	9	2020	0
L'Oreal Group	9	2008	0.01
Utrecht University	8	2009	0.01
Chonnam National University	7	2010	0
Jiangnan University	7	2020	0
University of Helsinki	7	2004	0
National Central University	7	2019	0
University of Milan	6	2012	0
Baylor College of Medicine	6	2013	0.04
University of Western Australia	6	2005	0
Icahn School of Medicine at Mount Sinai	6	2008	0.01
Harvard University	6	2002	0.03
Tehran University of Medical Sciences	6	2012	0.01
Beijing Technology and Business University	5	2019	0
Erasmus University Rotterdam	5	2006	0

However, Seoul National University demonstrated an intermediate centrality of 0, indicating a high publication count but limited collaborations with other institutions. Similarly, other institutions in South Korea, such as Chung Ang University and Kyung Hee University, followed a similar pattern. This phenomenon was consistent with the analysis of national collaborations in South Korea Furthermore, the University of Turku was the earliest in publishing articles in this field, indicating its pioneering role in the field.

#### 3.1.3 Author cooperation analysis

**Figure 5:**
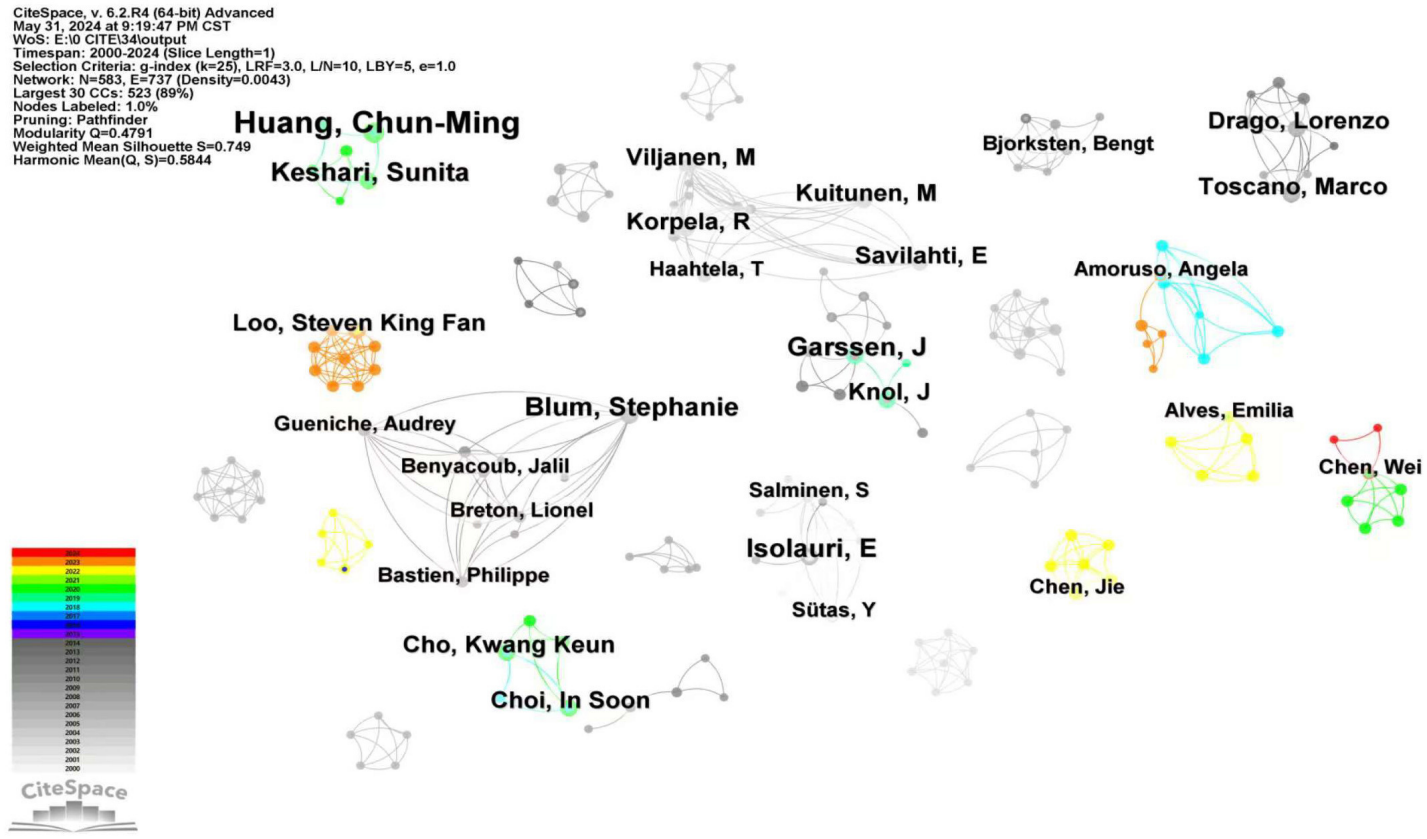
Author publication volume and collaboration analysis, using “Author” as the object of analysis in CiteSpace, we generated an analysis map of author cooperation and publications with 583 network nodes, 737 connections, and a density of 0.0043, displaying authors with a publication count of 3 or more. The size of the nodes corresponds to the frequency of article publications by each author. The lines between authors with relatively few collaborative instances have been omitted. The different colors represent distinct years, with each color corresponding to 1 year from 2024 (top) to 2000 (bottom), as indicated in the legend.

In Figure 5, the author with the most publications was Huang, Chun-Ming, who had seven related articles, and the publication years of the articles by Huang have been relatively cutting-edge. Compared to other research fields, the average number of articles per author has been relatively lower, indicating that authors in this field tend to focus on short-term research with few extendable topics. Additionally, research in this field has been relatively more dispersed due to the large number of participating authors and the relatively lower output of articles.

Furthermore, none of the authors have a centrality >0.1, indicating that authors have preferred independent research and have been less likely to collaborate with others. Objectively speaking, the lack of collaboration and communication is detrimental to the development and progress of the research field, which may also be a contributing factor to the low average number of articles per author. Therefore, authors need to engage in more extensive academic exchanges to expand their influence, share their strengths, and promote the development of the field. Table 3 presents the top 10 authors based on publication volume.

**Table 3:** Author publication volume and collaboration analysis.

**Authors**	**Frequency**	**Centrality**	**Years**
Huang, Chun-Ming	7	0	2019
Blum, Stephanie	5	0	2006
Garssen, J	5	0	2009
Isolauri, E	5	0	2000
Keshari, Sunita	5	0	2019
Choi, In Soon	4	0	2018
Drago, Lorenzo	4	0	2012
Knol, J	4	0	2010
Viljanen, M	4	0	2004
Toscano, Marco	4	0	2012

#### 3.1.4 Journal co-citation analysis

**Figure 6:**
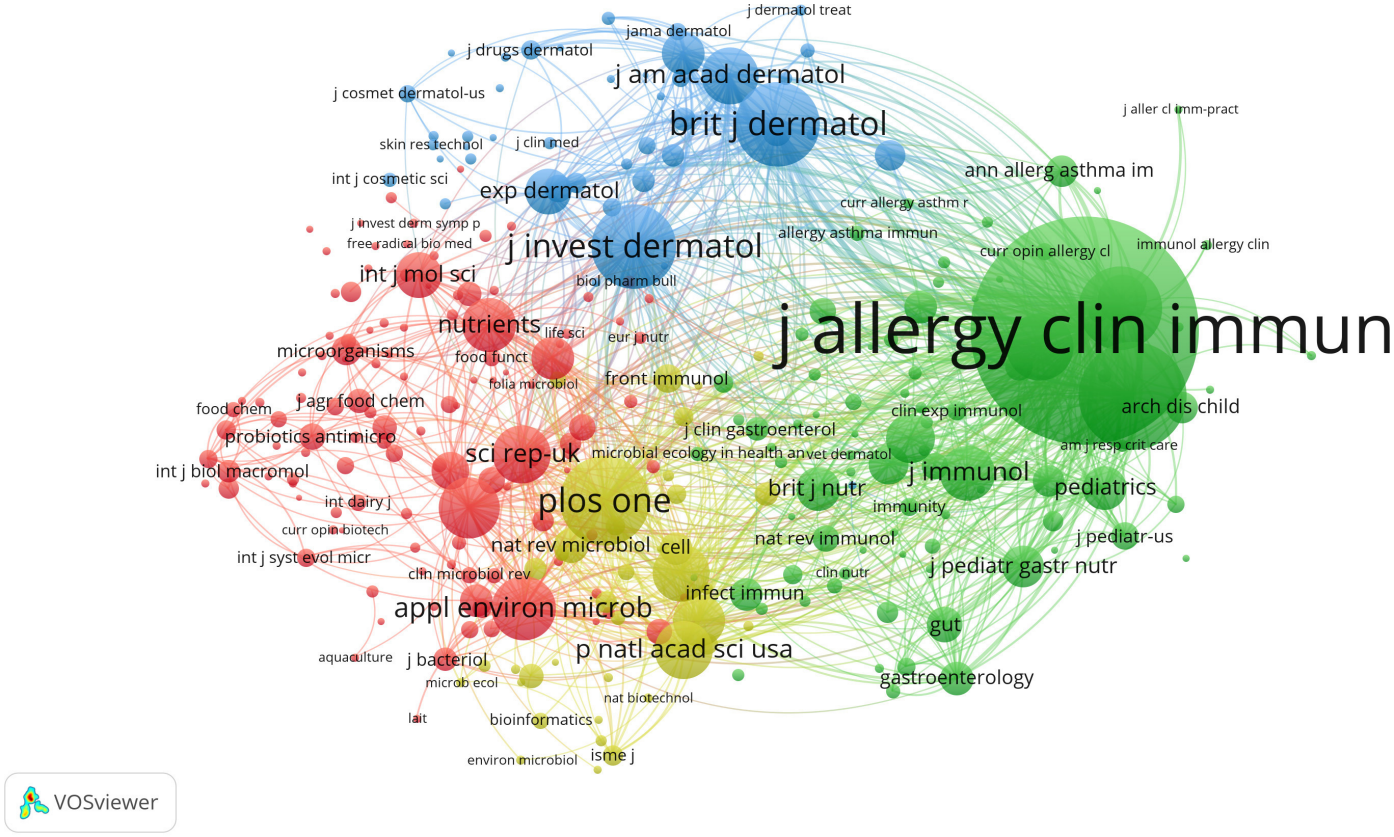
Analysis diagram of the journal co-citation network, each node represents a journal. Larger nodes indicate a greater number of published articles. Journals in the same color suggest that their articles in this field have similar themes and close relationships, and the lines connecting the nodes represent their co-citations within the same articles.

After conducting statistical analysis on publications and journal citations using VOSviewer (Figure 6), the field's three most authoritative core journals were identified. By examining information such as node size and citation counts in Figure 6 and Table 4, we could easily identify the journals with the highest number of published articles. Table 4 presents the top 20 journals in the field based on their citation counts. Upon comparing journal publications, citations, and impact factors, the core journals in the field of probiotics application for skin health were determined to be the *Journal of Allergy and Clinical Immunology, Clinical & Experimental Allergy*, and *Allergy*. The Journal of Allergy and Clinical Immunology has stood out with a relatively high number of published articles, the highest citation counts, average citation counts, and impact factor, rightfully establishing itself as the core journal in this field. While *Clinical & Experimental Allergy* had the highest number of published articles, its average citation counts ranked second. Noteworthy is the *British Journal of Dermatology*, which despite having only five published articles, ranked in the top 3 for its average citation count, indicating that although it had fewer articles, its published articles have had a more significant impact and contribution to the field's development.

**Table 4:** Journals with the top 20 citation numbers.

**Rank**	**Journals**	**Documents counts**	**Citations**	**Average citations**
1	Journal of Allergy and Clinical Immunology	19	4,314	227
2	Clinical and Experimental Allergy	23	2,914	127
3	Allergy	12	1,168	97
4	PLoS ONE	18	1,143	64
5	Pediatric Allergy and Immunology	13	788	61
6	Beneficial Microbes	19	632	33
7	British Journal of Dermatology	5	521	104
8	Scientific Reports	14	472	34
9	Experimental Dermatology	9	391	43
10	Journal of Clinical Gastroenterology	10	349	35
11	Probiotics and Antimicrobial Proteins	16	333	21
12	Annals of Allergy, Asthma and Immunology	*5*	309	62
13	Clinics in Dermatology	13	290	22
14	International Journal of Molecular Sciences	13	278	21
15	Nutrients	15	256	17
16	European Journal of Dermatology	5	224	45
17	Journal of Microbiology and Biotechnology	10	185	19
18	Frontiers in Microbiology	14	180	13
19	Journal of Applied Microbiology	5	164	33
20	Microbiome	5	158	32

### 3.2 Keywords cluster analysis, frontiers, and trends analysis

#### 3.2.1 Keywords cluster analysis

**Figure 7:**
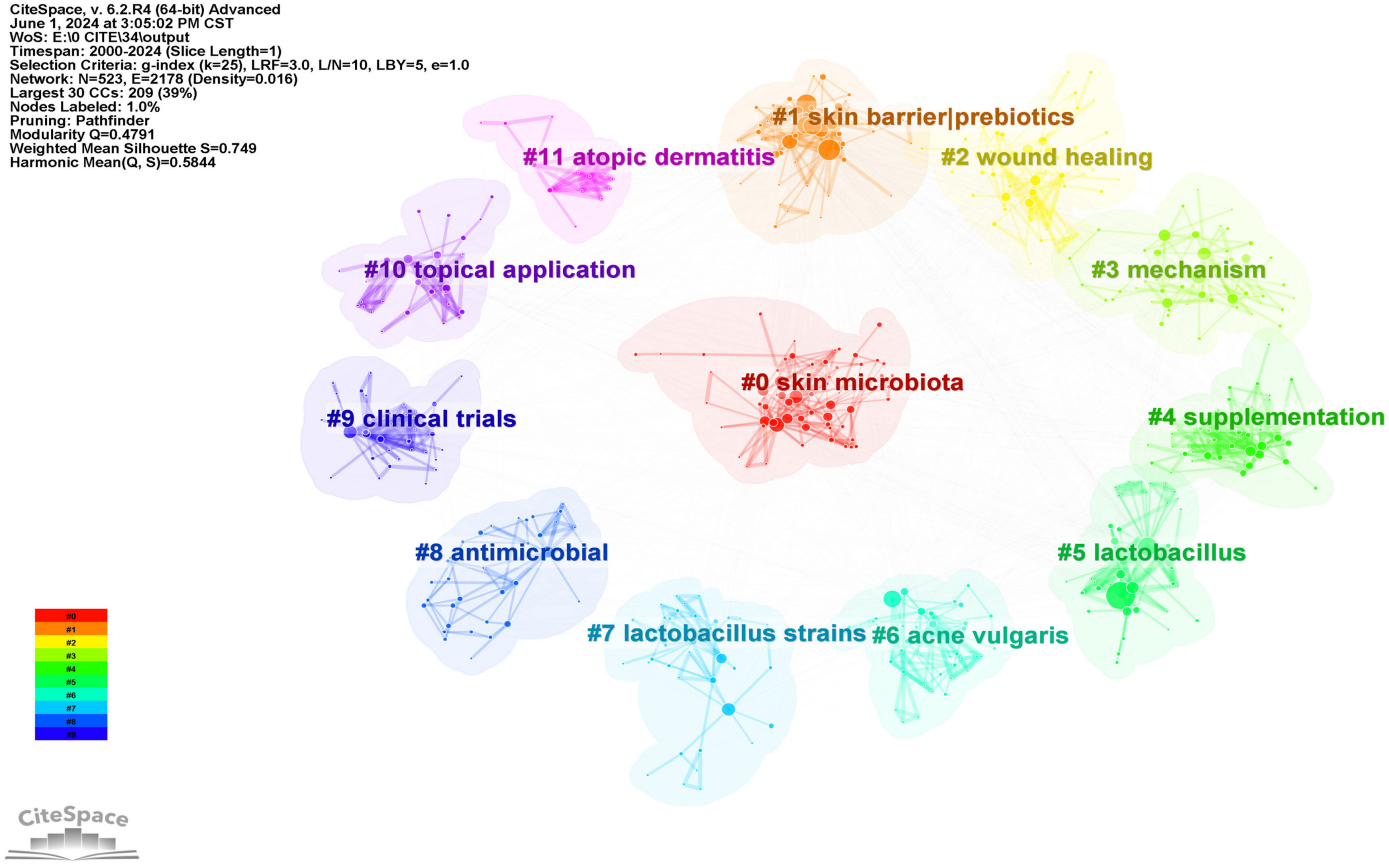
Analysis of keywords clustering, which was made by selecting the “cluster” option, and using the pathfinder algorithm to cut the connection lines to ensure the classification rationality of clusters. A total of 12 keyword clusters have been identified, with each cluster assigned a different color based on the time in the bottom left corner. The cluster names were derived from a set of representative keywords obtained using the LLR algorithm.

As can be seen from Figure 7, this study generated a total of 12 clusters, respectively: #0 skin microbiota (2016); #1 skin barrier|prebiotics (2012); #2 wound healing (2013); #3 mechanism (2016); #4 supplementation (2010); #5 lactobacillus (2006); #6 acne vulgaris (2011); #7 lactobacillus strains (2010); #8 antimicrobial (2014);#9 clinical trials (2007); #10 topical application (2007); #11 atopic dermatitis (2002). Each cluster covered specific keywords and research topics. The year indicates the average occurrence year of keywords within each cluster, shedding light on the proximity of the topics to the forefront of research.

The earliest cluster of research was on the application of probiotics to improve atopic dermatitis (#11 atopic dermatitis). It has been found that oral probiotics can significantly improve atopic dermatitis and other allergic diseases in infants, adults, pregnant women, and other patients. A large number of healthcare professionals and scientists have conducted numerous clinical trials to confirm the reliability of its therapeutic effects (#9 clinical trials) (Boyle et al., 2008). In addition, early research mainly focused at the genus level (#5 lactobacillus), such as Lactobacillus, Bifidobacterium, Streptococcus, etc. Subsequently, scientists have placed greater emphasis on studying the therapeutic effects of probiotics on skin diseases and improvements in skin health at the strain level (#7 lactobacillus strains) (Rosenfeldt et al., 2003; Panagiotou et al., 2023). In addition, in the early probiotics-related research on skin health, oral administration was the main method used, but later on, various forms of topical application of probiotics (#10 topical application) emerged, such as moisturizers (Im et al., 2018), hydrogels (Remaggi et al., 2023), textiles (Diep and Schiffman, 2023) and so on. In the middle stage, apart from atopic dermatitis, probiotics were extensively studied in relation to various new skin diseases represented by acne vulgaris (#6 acne vulgaris) and wound healing (#2 wound healing), and improvements in diseases such as hair loss (Yin et al., 2024), skin cancer (Friedrich et al., 2019), body odor (Yu et al., 2022), etc., were also associated with probiotic applications. Over time, the mechanisms by which probiotics improve skin health have been thoroughly explored, including enhancing skin barrier function (#1 skin barrier|prebiotics), resisting pathogens (Hafez et al., 2013) (#8 antimicrobial), stabilizing skin microbiota, increasing microbiota diversity (Truglio et al., 2024) (#0 skin microbiota), reducing inflammation (Lee K. S. et al., 2023), boosting antioxidant capacity (Lee B. K. et al., 2023), and regulating immunity (Yang et al., 2024) (#3 mechanism), etc.

#### 3.2.2 Analysis of research frontiers

In this study, the Bursts detection algorithm in Citespace software was utilized to generate a keyword burst map, illustrating the evolution of hotspots in the field. This table highlighted the top 30 keywords in the area, with associated burst intensity and duration information presented in Table 5.

**Table 5:** Top 30 keywords with the strongest citation bursts.

**Keywords**	**Strength**	**Burst begin years**	**Burst end years**
Dermatitis	5.83	2000	2012
Management	3.39	2004	2006
Intestinal microflora	5.32	2005	2010
Allergic disease	3.19	2007	2008
Placebo controlled trial	10.78	2008	2013
Infants	2.85	2008	2012
Hygiene hypothesis	2.77	2008	2010
Primary prevention	3.92	2010	2012
Lactobacillus GG	3.8	2012	2014
Adhesion	2.92	2012	2017
Strains	4.25	2014	2017
Supplementation	2.77	2014	2017
Regulatory t cells	3.76	2016	2019
Expression	5.02	2017	2019
Symptoms	3.51	2017	2021
Lactobacillus plantarum	2.82	2017	2021
Propionibacterium acnes	4.32	2018	2022
Infection	2.79	2018	2019
Skin lesions	2.61	2018	2020
Diversity	4.24	2019	2023
Chain fatty acids	4.14	2019	2021
Staphylococcus aureus	2.85	2019	2022
Prebiotics	7	2020	2022
Acne vulgaris	3.49	2020	2022
Skin microbiome	5.46	2021	2023
Wound healing	5.06	2021	2022
Severity	4.14	2021	2022
Oxidative stress	3.34	2021	2023
Microbiota	3.19	2022	2023
Antioxidant	2.66	2022	2023

Analysis of Table 5 reveals a consistent presence of hotspots in the field of probiotics application for skin health from 2000 to 2023. However, the distribution of keyword bursts over time has been uneven, with over two-thirds of the bursts originating in the latter half of the whole process. Additionally, more than half of the keyword bursts emerged in the past decade (starting from 2014), and these recent bursts have had shorter durations. This observation implies a rapid evolution within the field, with research directions continuously updated and enriched. Many of the keyword bursts have extended to the current period, signaling the ongoing advancement and development in this area.

Similar to keyword clustering, we can observe that research initially focused on the role of probiotics in improving allergic diseases, represented by dermatitis (“dermatitis”, “allergic disease”, “infants”), and related clinical trials (“placebo controlled trial”). Furthermore, in earlier periods, there already was an emphasis on the significant role that probiotics should play in global basic healthcare, as indicated by terms like “management,” “hygiene hypothesis,” and “primary prevention.” Around 2012, investigations about probiotic use for skin health progressed to the strain level, with a focus on specific strains like Lactobacillus GG. Moreover, the mechanisms by which probiotics promote skin health and treat skin diseases, such as through regulating immune responses (regulatory T cells), reducing inflammation, inhibiting pathogen adhesion (“adhesion”), modulating gene expression in skin cells (“expression”), producing short-chain fatty acids (“chain fatty acids”), enhancing skin microbiome diversity (“skin microbiome” and “microbiota”), and increasing antioxidant levels (“oxidative stress” and “antioxidant”), were gradually explored around 2012. Additionally, the function of probiotics in resisting pathogenic skin bacteria, such as Propionibacterium acnes and Staphylococcus aureus, has been increasingly investigated (“propionibacterium acnes,” “staphylococcus aureus,” and “infection”). Over time, new therapeutic effects of probiotics in treating various skin conditions, including “infections,” “skin lesions,” “acne vulgaris,” and “wound healing,” have been continuously discovered.

The current cutting-edge and focused keywords include “diversity,” “skin microbiome,” “oxidative stress,” “microbiota,” and “antioxidant,” reflecting the continuously evolving and dynamic nature in field of probiotics research for skin health.

## 4 Discussion

Bibliometrics is the study of scholarly publications that uses statistical data to establish correlations between works and explores the structural characteristics, favored trends, and frontiers of related research areas (Maggio et al., 2018). With the expansion of medical research literature, it has also been widely applied in the medical field to provide more cutting-edge information about drug treatments, diseases, and health science trends (Thompson and Walker, 2015). In this study, we conducted a literature search in WoSCC and included 709 original articles published that matched the topic. This paper summarized the trends and hotspots of probiotics application for skin health. It emphasized the importance of exploring the role of probiotics in skin health, which provides excellent value in promoting better skin health and treating skin diseases. Research related to the role of probiotics in skin health has shown significant growth. The annual publication output has increased from <3 to 102. By observing the publication trends, we find that the volume of research is expected to continue growing, with new scholars joining and new directions emerging. Therefore, this area requires a systematic summary to identify additional innovative research directions.

At the national level, countries with a centrality above 0.1 have been all developed countries, with most of them concentrated in regions such as Europe and North America, showing clear regional characteristics. This indicated that developed countries have placed greater emphasis on research obtained through inter-country cooperation and hold more authority in the field of probiotics application for skin health. The United States has had the highest number of publications and the second-highest centrality, making it the core country in this field, while Canada, despite ranking first in the intermediary centrality, has had fewer research outputs. At the institutional and author levels, research institutions in the field have tended to conduct relatively independent research, with collaborations being dispersed, except for the University of California System with a centrality above 0.1. Increased collaboration can integrate excellent research results and facilitate the generation of impactful academic outcomes. The core journals of probiotics application for skin health have been the *Journal of Allergy and Clinical Immunology, Clinical & Experimental Allergy*, and *Allergy*. *The Journal of Allergy and Clinical Immunology* has had a relatively higher number of articles and has ranked first in total citations, average citations, and impact factor, making it the undisputed core journal in this field.

Several probiotic species have been identified for their beneficial effects in managing skin disorders. The most commonly used probiotics in this context include *Lactobacillus plantarum, Lactobacillus acidophilus, Bifidobacterium longum*, and *Streptococcus thermophilus*. These probiotics have shown the ability to block the release of inflammatory cytokines, inhibit inflammatory mediators, and accelerate the recovery of skin barrier function. They have been used both topically and orally in the treatment of conditions such as acne, atopic dermatitis, and rosacea, helping to restore the balance of skin microbiota, reduce skin inflammation, and enhance overall skin health (Puebla-Barragan and Reid, 2021).

By summarizing the keywords clusters, emerging frontier terms, and burst terms related to the roles of probiotics in skin health, we could pinpoint the following trends:

1. In terms of research directions, there have been a noticeable shift from prioritizing clinical trials to emphasizing mechanism-based research.

2. Moreover, there has been a transition from the oral administration of probiotics to topical external treatments.

3. Additionally, the classification of probiotics has become increasingly refined, with a strong emphasis on the roles of specific strains.

4. The spectrum of skin conditions that probiotics can improve has continued to expand, ranging from an initial focus on dermatitis to encompass wound healing, acne, body odor, hair loss, moisturization, and anti-aging etc.

5. Importantly, research on how probiotics impact microbial communities or pathogens has gradually shifted its focus from the gut to the skin.

This article conducted an initial bibliometric analysis of publications concerning the role of probiotics in skin health to enhance understanding of the research landscape, highlight leading-edge topics, and identify emerging trends. This analysis aimed to offer a scientific summary and recommendations for the prudent utilization of probiotics in promoting skin health.

Despite the promising outcomes, it is important to acknowledge 4 primary limitations of this study:

1. The relatively limited volume of available literature in this specialized field, as we retrieved and screened literature using a double-blind method. This may lead to certain years lacking sufficient articles with threshold keywords, authors, institutions, or countries.

2. The constraints of bibliometric analysis may hinder a comprehensive description of all facets within this research domain. Due to software limitations, we only analyzed original English articles as they have complete citation records. CiteSpace's co-citation and co-occurrence functions can only analyze documents with complete records (Vinderola et al., 2024), and the above-mentioned studies are more detailed and reliable compared to other types.

3. Due to software design considerations, the visual analysis results may not effectively capture newly published high-quality literature when compared to older studies (Vinderola et al., 2024).

4. Given that we used TS retrieval rather than full-text retrieval, some relevant literature may have been missed. However, full-text retrieval results in a very high number of articles, significantly increasing unnecessary manual screening efforts.

## 5 Conclusion and perspectives

In this study, we employed bibliometric analysis tools such as CiteSpace and VOSviewer to systematically examine the research status concerning the role of probiotics in skin health from the perspectives of countries, institutions, authors, and journals. Furthermore, we summarized various prominent clusters and several major trends that have emerged throughout the progression of this research field since the 21^st^ century. We also identified current cutting-edge topics and made predictions based on these foundational findings regarding valuable potential directions for future development.

Looking ahead, the development directions in this field include research on probiotics for l external use, the formulation of probiotic and post-biotic products, exploration of the gut-skin axis and gut-brain-skin axis, elucidation of molecular mechanisms, bacterial-cell interactions, bacterial cross-talk, and the application of specific bacterial strains, etc. Furthermore, future research endeavors should prioritize addressing practical concerns such as antibiotic resistance, medical safety, potential pathogenicity, accessibility in global poverty-stricken areas, and food safety.

A critical perspective for future research is the topical application of probiotics, which is emerging as a key research focus for future developments in skin health. Given its potential importance, we conducted an in-depth analysis comparing the efficacy of traditional oral probiotics with topical applications. In this context, we examined several aspects of topical probiotics, including their efficacy, survival on the skin, stability, and safety.

Topical probiotics have shown promising results in directly influencing the skin microbiome, offering rapid and localized benefits, whereas oral probiotics primarily exert effects indirectly through systemic immune modulation. We believe that understanding these differences will help develop more effective skin health intervention strategies. Additionally, our study reviewed current research to address challenges associated with the survival, stability, and safety of probiotics applied topically.

### 5.1 Differences in the efficacy of topical vs. oral probiotics

Numerous studies have evaluated the efficacy of both topical and oral probiotics on skin health. Topical probiotics have demonstrated significant benefits in directly improving skin hydration and barrier function. For instance, a study applying probiotic ingredients to dry skin reported marked improvements, measured through bio-instrumental and proteomic analyses (Gueniche et al., 2010). In contrast, oral probiotics primarily work by modulating the gut microbiota, which indirectly benefits skin health. They can enhance gut barrier function and reduce systemic inflammation, subsequently improving skin conditions (Falholt Elvebakken et al., 2023). Notably, the direct action of topical probiotics often leads to faster and localized improvements, such as enhanced wound healing and reduced inflammation at the application site (Karimi et al., 2024).

Topical probiotics have demonstrated promising results in managing certain skin conditions. For example, a trial evaluating a topical probiotic cream for skin aging showed significant improvements in fine lines and uneven skin tone; however, the study did not compare its efficacy with oral probiotics (Falholt Elvebakken et al., 2023). Additionally, in atopic dermatitis, topical probiotics have been shown to improve SCORAD scores, suggesting that localized application may reduce inflammation and enhance skin barrier function, providing targeted therapeutic effects without the systemic side effects seen with oral probiotics (Chen, 2018). Further studies utilizing robust and well-designed research methodologies are needed to directly compare the efficacy of oral and topical probiotics using the same strains and to explore potential synergistic effects between both forms of administration.

### 5.2 Survival of probiotics on the skin

One of the main challenges to the efficacy of topical probiotics is their survival under the harsh conditions of the skin, which include low moisture, acidic pH, and immune defenses (Alziadi et al., 2020; Guéniche et al., 2010). However, innovative formulations have been developed to improve probiotic survival, such as encapsulating probiotics with specialized carriers that enhance their ability to colonize the skin (Karimi et al., 2024; Theodorou et al., 2024). Specific strains, such as Lactiplantibacillus plantarum, have shown good survival rates on the skin, particularly in moist environments like wounds or ulcers, further supporting their use in targeted therapeutic applications (Argañaraz Aybar et al., 2022).

### 5.3 Stability analysis

The stability of probiotics is a critical factor in determining their effectiveness in topical products. To ensure efficacy during storage and application, stability-enhancing techniques such as freeze-drying and multi-layer encapsulation are commonly used (Notay et al., 2017). For example, a formulation used for sensitive scalp conditions maintained high stability over time and successfully alleviated discomfort due to its robust composition (Alziadi et al., 2020) Stability remains particularly important for ensuring product efficacy over long-term storage and distribution periods.

### 5.4 Safety analysis

The safety of topical probiotics has been widely evaluated, with the majority of studies demonstrating that probiotic ingredients are generally safe for topical use, with no significant adverse skin reactions reported (Chen, 2018). For example, a study involving both healthy individuals and patients with atopic dermatitis found that topical probiotics were well tolerated and did not result in sensitization (Guéniche et al., 2010). However, in certain populations, such as immunocompromised individuals and patients with diabetes, the direct application of live bacteria to the skin may pose a risk of severe systemic infections (Doron and Snydman, 2015). Additionally, specific strains of probiotics have the potential to elicit allergic responses in susceptible individuals, highlighting the importance of comprehensive allergen screening during product development (Greenzaid et al., 2024). Moreover, strict measures for the preservation, identification, and quality control of probiotic strains are critical to ensuring their safe and effective widespread application. Genomic mutations can occur during successive generations of probiotic culture, which may introduce safety concerns (Sanders et al., 2010). Thus, while topical probiotics generally exhibit a favorable safety profile, careful strain selection, and rigorous quality control are paramount to minimizing potential risks.

In conclusion, research on the roles of probiotics in skin health has undergone significant growth and diversification, presenting promising opportunities for collaborative research and potential economic benefits.

## Data Availability

The original contributions presented in the study are included in the article/supplementary material, further inquiries can be directed to the corresponding authors.
