# Corrigendum: Probiotic effects on skin health: comprehensive visual analysis and perspectives

**DOI:** 10.3389/fmicb.2025.1583612

**Published:** 2025-03-12

**Authors:** Kexin Deng, Xiaofei Fan, Zhigen Yuan, Dian Li

**Affiliations:** ^1^Department of Orthopaedics, The Second People's Hospital of Hunan Province (Brain Hospital of Hunan Province), Changsha, Hunan, China; ^2^Department of Plastic and Reconstruction, The Third Xiangya Hospital of Central South University, Changsha, Hunan, China; ^3^Shandong Medical College, Jinan, Shandong, China; ^4^Department of Epidemiology, Los Angeles Fielding School of Public Health, University of California, Los Angeles, Los Angeles, CA, United States

**Keywords:** prebiotics, bibliometrics, skin, probiotics, bacteria

In the published article, there was an error in affiliation 3. Instead of “Shandong Medical College, Linyi, Shandong, China”, it should be “Shandong Medical College, Jinan, Shandong, China”.

The authors apologize for this error and state that this does not change the scientific conclusions of the article in any way. The original article has been updated.

